# Conventional and complementary cancer treatments: where do conventional and complementary providers seek information about these modalities?

**DOI:** 10.1186/s12913-018-3674-9

**Published:** 2018-11-14

**Authors:** Trine Stub, Sara A. Quandt, Thomas A. Arcury, Joanne C. Sandberg, Agnete E. Kristoffersen

**Affiliations:** 1 Department of Community Medicine, The National Research Center in Complementary and Alternative Medicine (NAFKAM), UiT The Arctic University of Norway, Sykehusveien 21, 9037 Tromsø, Norway; 2 Wake Forest School of Medicine, Department of Epidemiology and Prevention, Division of Public Health Sciences, Medical Center Boulevard, Winston-Salem, NC 27157 USA; 3 Wake Forest School of Medicine, Department of Family and Community Medicine, Medical Center Boulevard, Winston-Salem, NC 27157 USA

**Keywords:** Information seeking, Evidence–based medicine, Evidence -based information, Cancer care, Conventional health care providers, Complementary therapists, Complementary and alternative medicine

## Abstract

**Background:**

Both conventional health care providers and complementary therapists treat cancer patients. To provide effective treatment, both types of providers should to be familiar with their own as well as alternative types of treatment. Our aim was to compare how conventional health care providers (oncology doctors, oncology nurses, family physicians) and complementary therapists (acupuncturists, reflexologists, massage therapists) seek information about conventional and complementary cancer treatments.

**Method:**

This analysis was conducted on the basis of feedback from 466 participants. We used self-administered questionnaires in a cross-sectional study.

**Results:**

The majority of the medical doctors (96%) searched for evidence-based information regarding conventional cancer treatments. They gathered this information mostly from guidelines, which is considered best practice and is expected from Norwegian health personnel. Eighty-one percent of the nurses gather this information from evidence based resources such as UpToDate. Colleagues were asked for information by 58% of the medical doctors and 64% of the nurses. Moreover, 50% of the medical doctors and 57% of the nurses searched for evidence-based information about complementary cancer modalities. The acupuncturists gathered evidence-based information for both conventional (79%) and complementary (77%) modalities, followed by the reflexologists (54 and 54%, respectively) and massage therapists (54 and 52%, respectively). Nearly half of the acupuncturist (49%) asked a colleague for information.

**Conclusion:**

To provide safe cancer care, it is important that advice about complementary modalities is based on current and evidence-based evaluations. The majority of the medical doctors and nurses in this study sought information according to evidence-based medicine regarding conventional cancer treatments, and about half of them gathered evidence-based information about complementary cancer modalities. This was also true for the complementary therapists as they gathered information about complementary and conventional treatments from evidence-based evaluations. This demonstrates that since the term evidence-based medicine was first introduced in 1991, the approach has grown extensively and both conventional and complementary providers use this approach to seek information.

## Background

Many patients combine conventional and complementary therapies in cancer care [1]. Both conventional health care providers and complementary therapists treat cancer patients [2]. Therefore, to provide effective treatment, both types of providers need to be familiar with their own as well as alternative types of treatment. There are many sources of information about conventional and complementary modalities [3]. These vary from evidence-based evaluations [4] to less rigorous sources, such as the media and Internet that have less scientific basis. There are good evidence-based evaluations of both conventional and complementary modalities, and Norwegian health care personnel are expected to practice according to evidence-based medicine [5].

The most common definition of Evidence-Based Practice (EBP) is “the conscientious, explicit and judicious use of current best evidence in making decisions about the care of the individual patient. It means integrating individual clinical expertise with the best available external clinical evidence from systematic research” [4, 5]. The evidence, by itself, does not make the decision, but it can help support the patient care process. EBP is usually triggered by patient encounters, which generate questions about the effects of therapy, the utility of diagnostic tests, the prognosis of diseases, or the etiology of disorders. However, barriers often arise when knowledge is transferred to practice [6]. These barriers can be related to the quality of the actual research, characteristics of the health care provider, the work-organization, or the profession. Moreover, time needed to evaluating the research literature, may also impede the implementation of EBP [7].

In 2015, about 90.5 million people had cancer worldwide [8]. It caused about 8.8 million deaths, 15.7% of all human deaths. In the Nordic countries, cancer is the leading cause of mortality, accounting for 30% of all deaths. In Norway, 16,500 men and 14,000 women were diagnosed with cancer in 2013 [9]. Currently, approximately 242,000 Norwegians have a cancer diagnosis; one in four Norwegians will die as a result of cancer [9]. The three cancer types that take the most lives among men are lung cancer, prostate cancer and colon cancer. Among women, these are lung cancer, breast cancer and colon cancer [9].

Complementary and alternative medicine (CAM) is the term for medical products and practices that are generally not taught in medical schools, and are usually not offered in conventional (allopathic) medicine [10]. Complementary medicine includes treatments that are used along with conventional medical treatments, but not considered to be standard treatment, for example using acupuncture to help lessen some adverse effects of cancer treatment [11]. The use of complementary therapies is popular among cancer patients in Norway, and 34% of all cancer survivors reported CAM use in 2013 [12]. Patients often use these modalities to relieve pain, and to lessen symptoms of nausea and vomiting associated with chemotherapy or surgical anesthesia, in addition to reduce adverse effects of chemotherapy [2].

Using data obtained through a survey of conventional health care providers and complementary therapists, our goal was to compare information-seeking about conventional and complementary cancer care of [1] conventional health care providers (medical doctors [oncology doctors and family physicians] and oncology nurses), [2] providers with dual training, and [3] complementary therapists (acupuncturists, massage therapists and reflexologists/zone-therapists).

## Method

This study is based on data collected from a vanguard and main study [13]. The vanguard study was completed in October 2015, and provided information on the questionnaire face and content validity [14]. We conducted the main study from March to June 2016. The Regional Committees for Medical and Health Research Ethics (REC) reviewed the study protocol and found that there was no need for REC approval (2012/1318/REK Nord).

### Participants

**Fig. 1:**
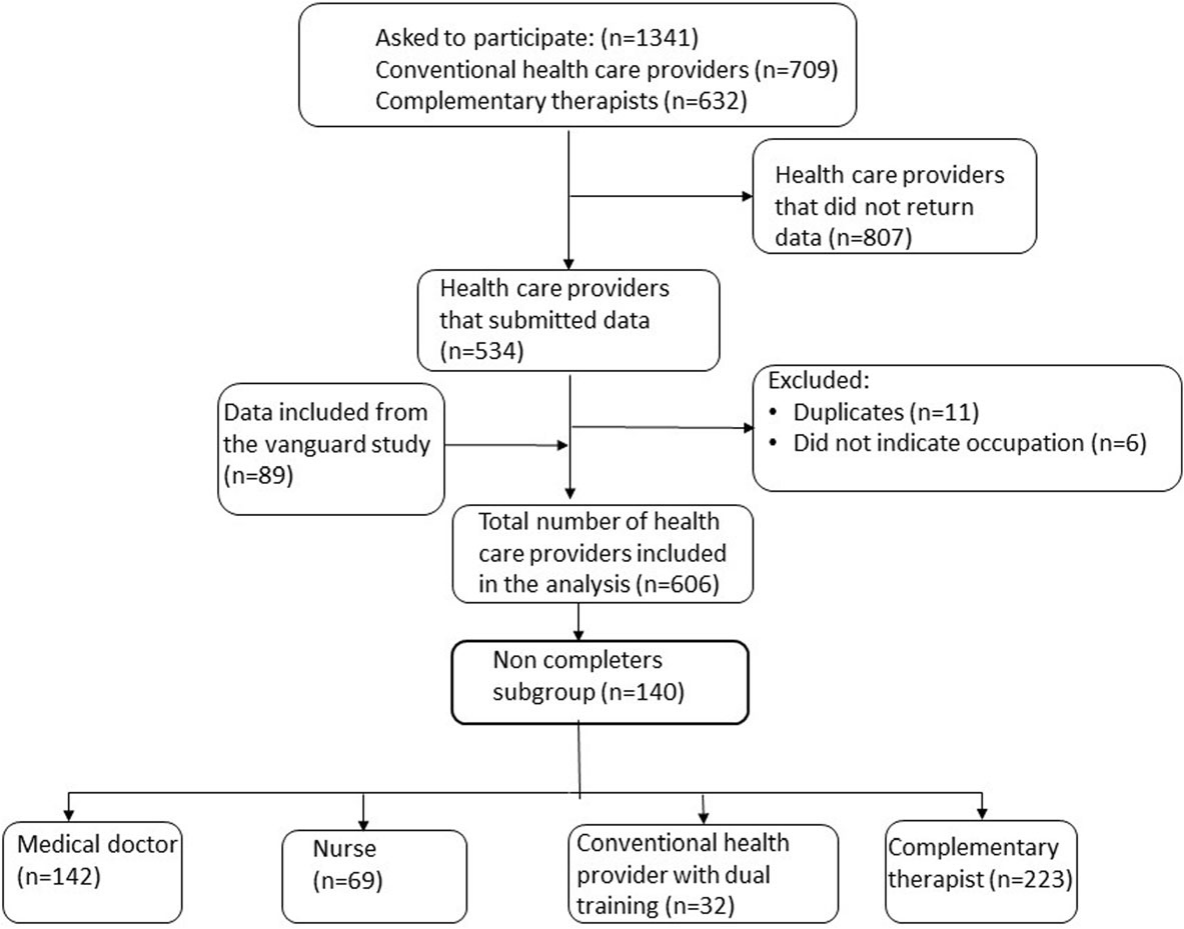
Flow chart of the inclusion process in this study

These were the criteria for inclusion in this study: Practicing oncology doctor, oncology nurse, family physician, or complementary therapist (acupuncturist, massage therapist, reflexologist/zone-therapist). In addition, clinical experience (current or previous) with cancer patients was required. The recruitment strategy ensured that all participants were members of a professional organization. When working on the main study, we sent e-mails or letters including questionnaires to 1341 people asking them to participate. We received feedback from 534. Due to duplication (*n* = 11) or lack of indicating profession (*n* = 6), 17 participants were excluded. The vanguard study contributed with data from 89 participants. These participants included 6 oncologists, 7 oncology nurses, 6 family physician and 70 complementary therapists. The number of exclusions due to non-completion amounted to 140 who returned incomplete questionnaires. The analysis was based on the final sample of 466 participants, which were distributed into four categories: Medical doctors (*n* = 142), nurses (*n* = 69), providers with dual training (*n* = 32) (education in conventional medicine as well as complementary modalities), and complementary therapists (*n* = 223) (Fig. 1).

### Recruitment

The oncology experts (doctors and nurses) were recruited through the University Hospital of North Norway (UNN), Tromsø, and the Vestfold Hospital Trust in Vestfold, Norway. The most used complementary modalities in Norway are massage therapy (11.9%), acupuncture 3.6%), naprapath (2.6%) and, zone therapy/reflexology (1.7%). Complementary therapists were therefore recruited through the Norwegian Association of Massage Therapists (NMF), the Norwegian Acupuncture Association (NAA) and the Norwegian association of Natural Medicine (NNH). Family physicians were recruited through the Norwegian Health Economics Administration (Helfo) webpage (http://helfo.no). We randomly selected names from the list and forwarded the questionnaire to them. Complementary therapists were recruited using lists provided by the NNA, the NMF and the NNH.

### Data collection

We collected data from self-administered questionnaires.

#### Questionnaire content

We developed the questionnaire based on information from a literature review [15]. We ended up using 65 questions divided into 7 themes (inclusion, 3 items; communication, 18 items;, risk in clinical practice, 14 items; perception about complementary and conventional treatment modalities, 12 items; information seeking about complementary therapies and conventional medicine, 6 items; demographics, 5 items; clinical practice or hospital work, 7 items).

#### Data collection procedures

Collection of data was based on Dillman survey procedures [16]. The participants were invited to participate in the study, in an electronic letter. This letter informed the recipients that they would receive a request to participate in an important study. One week later, e-mails were sent to all potential participants with a link to the online survey. After a week, an electronic “thank you” or reminder to complete the survey was sent to the selected providers using the same format that was used for the previous contact. Finally, 1 week later a reminder letter with a link to the survey was sent to the non-responders by e-mail. The physicians received the questionnaire by post, but with the option to complete the questionnaire either by post or email.

### Measures

Knowledge of complementary therapies was recorded in a matrix. This included: Acupuncture; homeopathy; hands on healing such as Reiki; Tai chi and qigong; aromatherapy; yoga; mindfulness; zone-therapy/reflexology; Chinese herbal medicine; other herbal medicine; chiropractic; osteopathy; and massage. The participants recorded their knowledge of each of the 13 modalities according to this scale: None (0), little (1), some (2), quite a bit (3), a great deal (4). All numbers were added, and the potential range was 0–52.

This was the question to measure how information about complementary modalities and cancer was sought: Do you ever seek information regarding complementary therapy and cancer? The response options were *no* or *yes*. There was one question to measure how information about conventional treatment was sought and a similar question about complementary therapies. Those who responded positively, had to specify where they sought this information. The choices were colleagues; family and friends; media; cancer organizations and cancer centers; guidelines; medical databases; on-line resources; professional conferences or seminars and professional associations. These sources of information were gathered into these 3 categories: (1) “Evidence-based literature” included these five elements: guidelines, medical databases (such as PubMed and Cochrane Library); on-line resources (such as UpToDate and BMJ BestPractice), professional conferences or seminars, and professional associations. These elements are similar to the Level of Evidence Table from Oxford Centre for Evidence–Based Medicine (levels 1–4) [17]. (2) “Non-evidence-based literature” which included family and friends; media and cancer centers or cancer organizations. (3) “Colleagues” was created as a third element as colleagues might reveal scientific evidence if an expert (level 5 on the OCEBM) and non-scientific if lacking this competence.

When stating their profession(s), the participants could use categories allowing them to report multiple professions, which were classified into four mutually exclusive “provider groups.” “Complementary therapists” included acupuncturists; massage therapists; or reflexologists/zone therapists who had no conventional training “Providers with dual training” included physicians and nurses, who provided complementary treatment, as well as complementary therapists who had other conventional training (e.g., physiotherapist).“Nurses” included oncology nurses who did not provide complementary treatment. “Medical doctors” included family physicians and oncologists who did not provide complementary treatment.

**Fig. 2:**
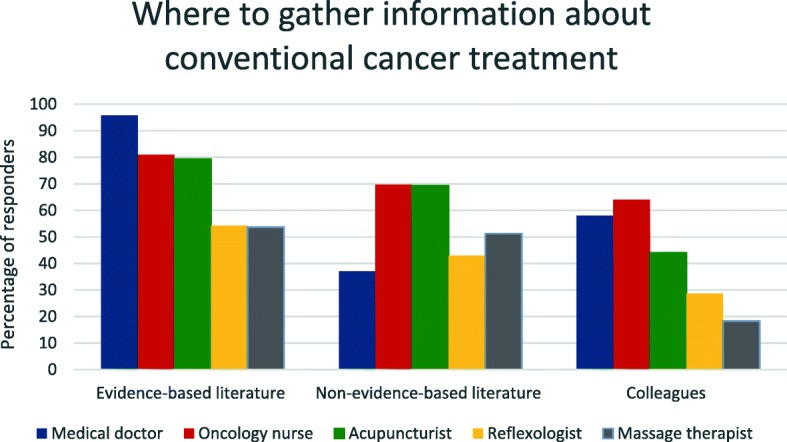
Where to gather information about conventional cancer treatment

**Fig. 3:**
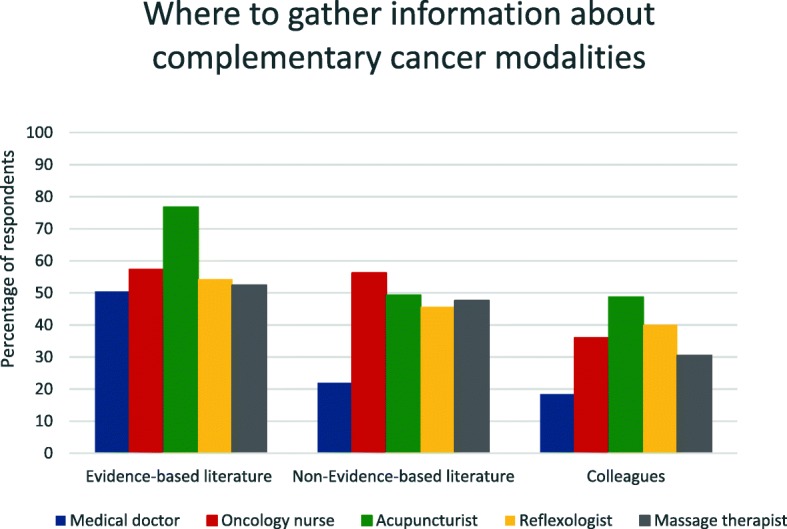
Where to gather information about complementary cancer modalities

When analyzing “Where to gather information about conventional cancer treatment” and “Where to gather information about complementary cancer modalities” (Figs. 2 and 3), we decided to merge the profession categories “Oncologists” and “Family physicians” into “Medical doctors”, leaving us with five categories. The aim was to investigate whether there was any difference related to profession when the participants sought for information. The categories “Medical doctors”, “Nurses”, “Acupuncturists”, “Massage therapists” and “Reflexologists” were not mutually exclusive. Hence, it is possible that a participant might occur in more than one category.

### Statistical analysis

We calculated descriptive statistics (counts, percentages) for each profession and each provider group. We analyzed the comparison of characteristics of the various providers and their practice. When analyzing the differences in categorical variables, we used the Pearson chi square test or the Fisher’s exact test. When comparing continuous variables, we used the One way Anova test. We set the significance level as *p* < 0.05 with no adjustment for multiple comparisons. For the analysis we used SPSSV.24.0 for Windows.

## Results

### Demographics

The professions reported in the study included oncologists (*n* = 27), family physicians (*n* = 118), oncology nurses (*n* = 89), acupuncturists (*n* = 150), massage therapists (*n* = 82), and reflexologists/zone-therapists (*n* = 35) (Table 1). They were grouped into medical doctor (*n* = 142, 30.5%), nurse (*n* = 69, 14.8%), provider with dual training (*n* = 32, 6.9%) and complementary therapist (*n* = 223, 47.6%) (Table 1).

**Table 1:** Characteristics of the participants (*n* = 466)***

	Total (*n* = 466)		Medical doctor (*n* = 142)		Nurse (*n* = 69)		Providers with dual training (*n* = 32)		Complementary therapist (*n* = 223)		*p*-value
	n	(%)	n	(%)	n	(%)	n	(%)	n	(%)	
Gender											< 0.001*
Male	108	(27.5)	69	(51.5)			3	(12.5)	36	(19.3)	
Female	285	(72.5)	65	(48.5)	48	(100)	21	(87.5)	151	(80.7)	
Missing	73		8		21		8		36		
Age, years											< 0.001**
Mean age	373	48.7	127	45.4	45	51.2	24	52.2	177	50.1	
Missing	93		15		24		8		46		
Education											< 0.001^
Compulsory	2	(0.5)							2	(1.1)	
Middle level	33	(8.4)							33	(17.6)	
University up to 4 years	112	(28.4)			23	(46.9)	11	(44.0)	78	(41.7)	
University more than 4 years	235	(59.5)	123	(91.1)	26	(53.1)	14	(56.0)	73	(39.0)	
PhD	13	(3.3)	12	(8.9)					1	(0.5)	
Missing	71		8		20		7		36		
Profession*											
Oncology doctor	27	(5.8)	27	(100)							
Family physician	118	(25.3)	116	(99.1)			1 ^a^	(1.8)			
Oncology nurse	89	(19.1)			69	(77.5)	20	(22.5)			
Acupuncturist	150	(32.2)					25	(16.7)	125	(83.3)	
Massage therapist	82	(17.6)					6	(7.3)	76	(92.7)	
Reflexologist/zonetherapist	35	(7.5)					1	(2.9)	34	(97.1)	
Clinical practice											< 0.001^
Full time health provider	287	(72.1)	121	(89.6)	38	(77.6)	18	(72.0)	110	(58.2)	
Part time health provider	92	(23.1)	11	(8.1)	10	(20.4)	5	(20.0)	66	(34.9)	
Other (students or retired persons)	19	(4.8)	3	(2.2)	1	(2.2)	2	(8.0)	13	(6.9)	
Missing	68		7		20		7		34		
Patient visits per week											< 0.001*
1–19 patients	131	(33.8)	10	(7.6)	27	(57.4)	4	(16.0)	90	(48.6)	
20–39 patients	121	(31.2)	28	(21.4)	17	(36.2)	5	(20.0)	71	(38.4)	
40 or more patients	136	(35.1)	93	(71.0)	3	(6.4)	16	(64.0)	24	(13.0)	
Missing	78		11		22		7		38		
Cancer patient visits per week											< 0.001^
1–19 cancer patients	361	(92.1)	125	(92.6)	31	(64.6)	23	(92.0)	182	(98.9)	
20 and more patients	31	(7.9)	10	(7.4)	17	(35.4)	2	(8.0)	2	(1.1)	
Missing	74		7		21		7		39		
Location											0.005*
Rural area	118	(29.7)	56	(41.5)	7	(14.6)	3	(12.0)	52	(27.5)	
Small city. Village (up to 50.000 inhabitants)	153	(38.5)	44	(32.6)	23	(47.9)	12	(48.0)	74	(39.2)	
Large city (> 50.000 inhabitants)	126	(31.7)	35	(25.9)	18	(37.5)	10	(40.0)	63	(33.3)	
Missing	69		7		21		7		34		

* Pearson’s chi-square test

** One way Anova test

^Fisher’s exact test

*** Due to multiple response on one or more variables, the analyzed numbers do not always add up to the total number

^a^ These add to > 32 because providers have more than one area of training

Medical doctors were significantly younger than the other groups (*p* < 0.001). The groups also differed significantly in education, with medical doctors having the highest proportion with more than 4 years of university training (*p* < 0.001).

Most medical doctors and nurses worked full time (90 and 78%, respectively), compared with complementary therapists (58%) (*p* < 0.001). Medical doctors and providers with dual training reported more patient visits per week (*p* < 0.001). The groups differed in practice locations (*p* = 0.005), with the largest proportion of medical doctors (42%) located in rural areas. The largest proportion of nurses (48%), providers with dual training (48%) and complementary therapists (39%) worked in small towns (Table 1).

### Knowledge about complementary therapies

Complementary therapists and providers with dual training claimed more total knowledge about the 13 listed complementary modalities than conventional health care providers (*p* < 0.001). Providers with dual training had the highest mean score (mean 27.1, range 17–39, 95%CI 24.77–29.51). Complementary therapists had a mean score of 26.2 (range 10–48, 95%CI 25.24–27.16); the medical doctors and the oncology nurses had the lowest mean scores, 16.8 (range 0–42, 95%CI 15.38–18.28) and 18.5 (range 4–31, 95%CI 16.65–19.65), respectively.

### Where to seek information about conventional and complementary cancer treatment

#### Conventional cancer treatment

Most medical doctors (96%, *n* = 137) gathered information about conventional cancer treatment from evidence-based literature (mostly from guidelines), compared with oncology nurses (81%, *n* = 72) acupuncturists (79%, *n* = 119), reflexologists (54%, *n* = 19)) and massage therapists (54%, *n* = 44). Seventy percent (*n* = 62) of nurses gathered information from non-evidence based literature, compared with acupuncturists (69%, *n* = 104), massage therapists (51%, *n* = 42), reflexologists (43%, *n* = 15) and medical doctors (37%, *n* = 53). Colleagues were asked for information by 58% of the medical doctors and 64% of the nurses. As some of the individuals occurred in more than one provider group (see Table 2), between-group analyses could not be performed (Fig. 2).

**Table 2:** Distribution of providers with more than one area of training

	Medical doctor (MD)	Oncology nurse	Acupuncturist	Massage therapist	Reflexologist
Medical doctor (MD)	142		1		
Oncology nurse					
Acupuncturist					
Massage therapist					
Reflexologist					
Oncology nurse		73	10	1	1
Medical doctor (MD)					
Acupuncturist					
Massage therapist			4		
Reflexologist					
Acupuncturist			120	4	2
Medical doctor (MD)					
Oncology nurse					
Massage therapist			8		
Reflexologist				1	
Massage therapist				60	4
Medical doctor (MD)					
Oncology nurse					
Acupuncturist					
Reflexologist					
Reflexologist					27
Medical doctor (MD)					
Oncology nurse					
Acupuncturist					
Massage therapist					

Many of the participants had more than one area of training (Table 2). For example, 10 oncology nurses had additional training in acupuncture and four of these were also trained as massage therapists. Some of them had up to 3 types of training.

#### Complementary cancer modalities

Acupuncturists were the provider group that most commonly searched for evidence-based literature about complementary cancer modalities (77%, *n* = 115), followed by reflexologists (54%, *n* = 19) and massage therapists (52%, *n* = 43). Moreover, 50% (*n* = 71) of the medical doctors searched for evidence-based literature, so did 57% (*n* = 51) of the oncology nurses. Fifty-six percent (*n* = 50) of the nurses also gathered information from non-evidence based literature, followed by the acupuncturists (49%, *n* = 74), massage therapists (48%, *n* = 39), reflexologists (46%, *n* = 16) and medical doctors (22%, *n* = 31). Nearly half of the acupuncturist (49%) asked a colleague for information. (Fig. 3). As some of the individuals occurred in more than one group, no between-group analyses could be performed.

## Discussion

Generally, each professional group searched for evidence-based information about conventional and complementary cancer treatment to a larger degree than relying on potentially non-evidence-based information and information from colleagues. The majority of the medical doctors and oncology nurses searched for evidence-based information regarding conventional cancer treatments. One important purpose of evidence-based medicine is to raise awareness of which sources of information medical and health-related decisions are based on. Practicing evidence-based medicine prevent therefore that decision are random based [18]. The participants in this study gathered this information mostly from guidelines, which are best practices and expected from Norwegian health personnel [18–20]. Fifty-percent of the medical doctors and 60% of the nurses gathered this information from evidence-based resources. This is in contrast to a survey with medical students and faculty members in New York State, which found that physicians were aware of different complementary modalities, but that they failed to check adverse effects and interactions of supplements/herbs in reference texts [25]. This demonstrates that the approach has grown extensively and both conventional and complementary providers use this approach to seek information [20].

The results of the questions used about knowledge of complementary modalities may or may not reflect actual knowledge of the respondents. The differences among the participants may reflect the participants’ openness towards complementary modalities in general or other factors. To provide effective and safe cancer care, it is, however, important that conventional health care personnel have knowledge about complementary modalities. This does not mean that they have to be an expert to have meaningful conversations about complementary medicine with their patients [21]. Moreover, Kemper and colleagues [22] performed a randomized crossover trial where participants were invited to take part in an Internet-based curriculum on health care providers’ knowledge, confidence and clinical practices related to herbs and dietary supplements. These researchers found that knowledge, attitudes and self-reported practice were significantly improved through this program.

More than three quarters of the acupuncturists and more than half of the reflexologists and massage therapists in our study gathered evidence-based information about conventional and complementary cancer modalities. This is new knowledge about complementary therapists in Norway, demonstrating that the majority of these providers practice evidence-based treatment. The result is in line with Grey et al. [23], who found that complementary therapists had considerable interest in further education in cancer care and that they have an important role to play in the post diagnostic care for women with cancer.

### Practical implications

Cancer patients want to discuss the use of complementary modalities with their conventional health care providers [24]. Therefore, medical doctors and nurses need to be familiar with alternative and complementary (CAM) treatments. This study shows that the majority of the participants used evidence-based sources when seeking information about complementary modalities in cancer care. However it is room for further improvement. Therefore, evidence-based resources, such as CAM Cancer (http://www.cam-cancer.org), are available, and may be useful for discussing the pros and cons of complementary modalities with cancer patients.

### Limitations

This analysis should be interpreted in light of its limitations. The response rate may be a threat to the generalizability of the findings, because the non-responders may differ from those who responded [14]. However, the findings for conventional and complementary cancer care information-seeking are in accordance with other studies [15, 25], which suggests that the nonresponse bias probably imposes no major threat to the validity of the results [26].

## Conclusion

To provide safe cancer care, it is important that advice about complementary modalities is based on current and evidence-based evaluations. The majority of both conventional and complementary providers in this study gathered information from such evaluations. This demonstrates that the evidence-based approach has grown extensively to the best for patients and decision makers.

## Abbreviations

CAM: Complementary and alternative medicine
EBP: Evidence-based practice
REC: Committees for medical and health research ethics

## References

[1] National Center for Health Statistics. National Health Interview Survey (NHIS):2007 data release 2007 07.10. Available from: http://www.cdc.gov/nchs/nhis/nhis-2007_data_release.htm. [Cited 10 June 2015].

[2] Deng GE, Frenkel M, Cohen L, Cassileth BR, Abrams DL, Capodice JL (2009). Evidence-based clinical practice guidelines for integrative oncology: complementary therapies and botanicals. Soc Integr Oncol.

[3] Institute of Medicine (2011). National, clinical practice guidelines we can trust.

[4] Sackett DL, Rosenberg WC, Gray JAM, Haynes RB, Richardson WS (1996). Evidence based medicine: what it is and what it isn't. BMJ.

[5] Nortvedt MW, Jamtvedt G, Graverholt B, Nordheim LV, Reinar LM. Jobb kunnskapsbasert!: en arbeidsbok. English: Work Evidencebased!: A Workbook Oslo: Akribe; 2012.

[6] Montori VM, Guyatt GH (2008). Progress in evidence-based medicine. JAMA.

[7] Greenhalgh T (1997). How to read a paper: papers that summarise other papers (systematic reviews and meta-analyses). BMJ.

[8] GBD 2015 Disease and Injury Incidence and Prevalence Collaborators (2016). Global, regional, and national incidence, prevalence, and years lived with disability for 310 diseases and injuries, 1990–2015: a systematic analysis for the Global Burden of Disease Study 2015. Lancet.

[9] Norwegian Institute of Public Health (NIPH). Cancer mortality in Norway - fact sheet Oslo: Norwegian institute of Public Health; 2016. Available from: https://www.fhi.no/en/mp/chronic-diseases/cancer/cancer-mortality-in-norway%2D%2D-fact-s/. [Updated 18.04.2016; Cited 01.04 2017]

[10] Fønnebø Vinjar (2015). Practitioners of complementary and alternative medicine should value their strengths. Focus on Alternative and Complementary Therapies.

[11] National Cancer Institute at the National Institutes of Health. Complementary and Alternative Medicine Bethesda, MD National Cancer Institute; 2015 [Cited 23 06 2017]. Available from: https://www.cancer.gov/about-cancer/treatment/cam.

[12] Kristoffersen A, Norheim AJ, Fønnebø V (2013). Complementary and alternative medicine use among Norwegian Cancer survivors: gender-specific prevalence and associations for use. Evid Based Complement Alternat Med.

[13] Stub T, Quandt SA, Arcury TA, Sandberg JC, Kristoffersen AE (2017). Complementary and conventional providers in cancer care: experience of communication with patients and steps to improve communication with other providers. BMC Complement Altern Med.

[14] Bordens KS, Abbott BB (2002). Research Design and Methods. A process approach.

[15] Stub T, Quandt SA, Arcury TA, Sandberg JC, Kristoffersen AE, Musial F (2016). Perception of risk and communication among conventional and complementary health care providers involving cancer patients’ use of complementary therapies: a literature review. BMC Complement Altern Med.

[16] Dillman DA, Smuty JD, Christian LM (2009). Internet, mail and mixed-mode surveys. The tailored design method.

[17] OCEBM Levels of Evidence Working Group. The Oxford 2011 levels of evidence: Oxford Centre of Evidence-Based Medicine; 2011. Available from: http://www.cebm.net/index.aspx?o=5653. [Cited 20 06 2017]

[18] Sackett DL, Straus S, Richardson S, Rosenberg W, Hayens B (2000). Evidence-based medicine: how to practice and teach EBM.

[19] Guyatt G, Cairns J, Churchill D (1992). Evidence-based medicine: a new approach to teaching the practice of medicine. JAMA.

[20] Bondevik H, Engebretsen E, Roos I, Tønnesen J (2017). Innføring av ‘kunnskapsmedisin’ i norsk medisinsk diskurs. English: introduction of evidence-based medicine in Norwegian medical discourse. Sann opplysning? Naturvitenskap i nordiske offentligheter gjennom 400 år English: true information? Natural science in Nordic public institutions.

[21] Silverstein DD, Spiegel AD (2001). Are physicians aware of the risks of alternative medicine?. J Community Health.

[22] Schofield Penelope, Diggens Justine, Charleson Catherine, Marigliani Rita, Jefford Michael (2010). Effectively discussing complementary and alternative medicine in a conventional oncology setting: Communication recommendations for clinicians. Patient Education and Counseling.

[23] Kemper KJ, Amata-kynvi A, Sanghavi D, Whelan JS, Dvorkin L, Woolf A (2002). Randomized trial of an internet curriculum on herbs and other dietary supplements for health care professionals. Acad Med.

[24] Gray RE, Fitch M, Saunders PR, Wilkinson A, Ross CP, Franssen E (1999). Complementary health practitioner’s attitudes, practices, and knowledge related to women’s cancers. Cancer Prevent Control.

[25] Verhoef MJ, Trojan L, Armitage GD, Carlson L, Hilsden RJ (2009). Complementary therapies for cancer patients: Assessing information use and needs. Chronic Dis Can.

[26] Halbesleben JRB, Whitman MV (2013). Evaluating survey quality in health services research: a decision framework for assessing nonresponse Bias. Health Serv Res.

